# Venoarterial Extracorporeal Membrane Oxygenation Rescue for Catastrophic Grade 3 Bone Cement Implantation Syndrome in a Patient With Aortic Valve Stenosis and Moderate Pulmonary Hypertension: A Case Report

**DOI:** 10.7759/cureus.101514

**Published:** 2026-01-14

**Authors:** Hiroaki Kume, Nobuyuki Nosaka, Rie Yasumura

**Affiliations:** 1 Department of Intensive Care Medicine, Graduate School of Medical and Dental Sciences, Institute of Science Tokyo, Tokyo, JPN; 2 Division of Anesthesiology, Kawasaki Saiwai Hospital, Kawasaki, JPN; 3 Division of Anesthesiology, National Hospital Organization Tokyo Medical Center, Tokyo, JPN

**Keywords:** bone cement implantation syndrome, cardiac arrest, pulmonary embolism (pe), right heart failure, veno-arterial extracorporeal membrane oxygenation (va ecmo)

## Abstract

Bone cement implantation syndrome (BCIS) is a rare but potentially fatal complication of cemented arthroplasty, characterized by hypoxia, hypotension, and/or cardiovascular collapse. Although right ventricular failure is considered a key mechanism, its pathophysiology - potentially driven by pulmonary microembolization, complement activation, and the release of histamine - remains poorly defined. Due to the high mortality of severe BCIS, evidence regarding optimal management is limited.

An 80-year-old woman with moderate-to-severe aortic valve stenosis and moderate pulmonary hypertension (PH) underwent total hip arthroplasty (THA). Five minutes after bone cement implantation, she developed sudden, profound cardiopulmonary collapse. Pulmonary embolism (PE) was suspected but excluded by transesophageal echocardiography (TEE) and computed tomography (CT) pulmonary angiography. Venoarterial extracorporeal membrane oxygenation (VA-ECMO) was promptly initiated, resulting in hemodynamic stabilization. She was successfully weaned from ECMO and discharged from the Intensive Care Unit (ICU) on Day 23 and from the hospital on Day 52, without neurological complications.

This case highlights that prompt recognition of BCIS and immediate VA-ECMO initiation may be vital for achieving favorable outcomes in catastrophic BCIS, in selected cases managed at specialized centers.

## Introduction

Bone cement implantation syndrome (BCIS) is a rare but potentially fatal complication of bone cement use in orthopedic surgery. It is characterized by hypoxia, hypotension, and/or cardiovascular collapse [1,2]. Despite the establishment of diagnostic criteria, many cases remain underrecognized, and Grade 3 BCIS is frequently fatal.

We report a rare case of successfully resuscitated Grade 3 BCIS, complicated by cardiac arrest and ventricular fibrillation, in a patient with severe aortic stenosis (AS) and pulmonary hypertension (PH), highlighting the importance of early recognition, multidisciplinary preparation, and rapid extracorporeal cardiopulmonary resuscitation (ECPR).

## Case presentation

The classification of BCIS is provided in Table 1 and is characterized by hypoxia, hypotension, and/or cardiovascular collapse [1,2].

**Table 1:** Classification of BCIS Source: [1,2] BCIS, Bone cement implantation syndrome

Grade	Definition
Grade 1	Moderate hypoxia with peripheral oxygen saturation (SpO₂) < 94%, or low blood pressure with > 20% reduction in systolic pressure
		Grade 2	Severe hypoxia with SpO₂ < 88%, or low blood pressure with > 40% reduction in systolic pressure, or sudden loss of consciousness
Grade 3	Cardiovascular collapse requiring intensive care (arrhythmia, cardiogenic shock, or cardiac arrest)		

Preoperative course

An 80-year-old female (height: 159.2 cm and weight: 53.2 kg) presented after a fall and was scheduled for total hip arthroplasty (THA) the following day. Her comorbidities included atrial fibrillation (AF), moderate-to-severe AS (V max 3.9 m/s, mean gradient 34 mmHg, valve area 0.5 cm²), and moderate PH (right ventricular systolic pressure (RVSP) 52 mmHg on echocardiography). She had no clinical evidence of heart failure. The American Society of Anesthesiologists Physical Status (ASA-PS) classification was Class Ⅲ [3]. After a multidisciplinary discussion between the anesthesiology and cardiology teams, THA under general anesthesia was planned, with continuous hemodynamic monitoring.

Intraoperative course and cardiac arrest

During surgery, the mean arterial pressure (MAP) was maintained at approximately 65 mmHg using continuous norepinephrine infusion. Five minutes after bone cement implantation, the patient developed an abrupt decrease in end-tidal CO₂ (EtCO₂) and blood pressure, refractory to vasopressors. Arterial pressure monitoring revealed a loss of pulse pressure, indicating cardiac arrest.

The surgical procedure was immediately halted, and the patient was repositioned from lateral to supine. Intravenous epinephrine was administered, and cardiopulmonary resuscitation (CPR) was initiated 10 minutes after the diagnosis of cardiac arrest. Acute right heart failure, secondary to massive pulmonary embolism (PE), was initially suspected. Transesophageal echocardiography (TEE) during CPR revealed aortic valve closure and marked right heart dilation, which suggested acute right ventricular failure. However, no emboli were observed in the pulmonary artery.

Resuscitation and ECMO initiation

Venoarterial extracorporeal membrane oxygenation (VA-ECMO) was established by a cardiologist via the right femoral approach within 30 minutes of CPR initiation. ECMO was initiated at a pump speed of 2,500 rpm, a flow rate of 3 L/min, and a sweep gas flow of 3 L/min. Despite ongoing bolus and continuous infusions of norepinephrine and epinephrine, spontaneous circulation was not achieved until ECMO initiation. The rhythm converted from ventricular fibrillation to sinus rhythm after amiodarone administration under ECMO support.

**Figure 1:**
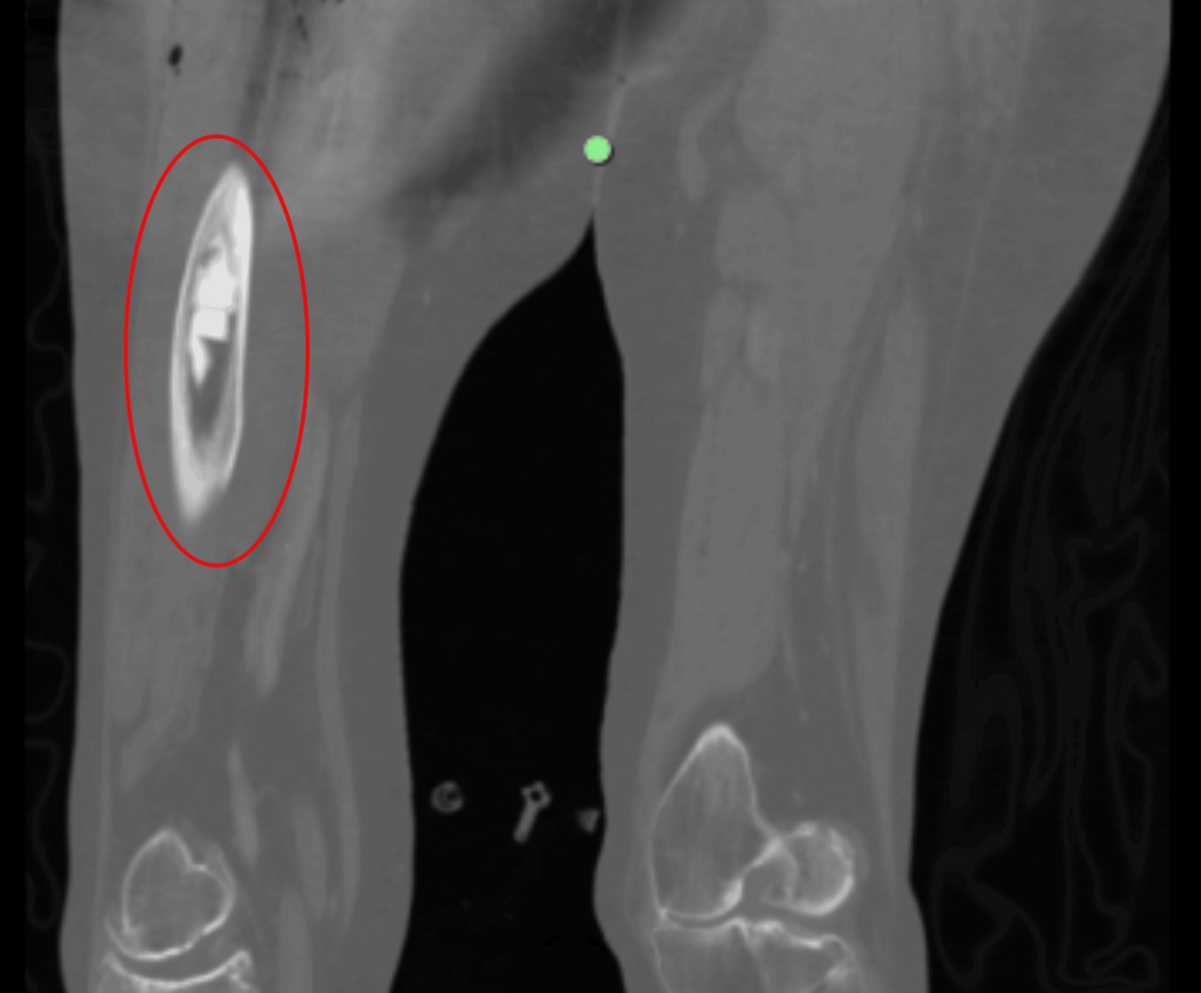
Postoperative CT of patient’s foot The red circle shows cement extending beyond the medullary cavity. CT, Computed tomography

The patient was transferred to the Intensive Care Unit (ICU) under stable ECMO flow. Computed tomography (CT), performed immediately after admission, showed poor peripheral pulmonary enhancement but no definitive embolus. Bone cement was noted to extend beyond the medullary cavity, as shown in Figure 1.

Postoperative course

Follow-up imaging, including serial CT scans performed on the day of onset and on Day 1, as well as TEE conducted in the ICU, revealed no evidence of PE or other thromboembolic disease. Adjunctive therapies, including intra-aortic balloon pumping (IABP) and inhaled nitric oxide (iNO), were initiated to support right ventricular function. Pulmonary arterial pressure remained stable throughout the ICU stay. The patient was successfully weaned off VA-ECMO on Day 9, IABP on Day 13, and iNO on Day 14. Extubation was achieved on Day 16. Tube feeding was started on Day 9, and a dysphagia diet was able to be started on Day 36. She was discharged from the ICU on Day 23 and from the hospital on Day 52, without neurological sequelae. Continuing rehabilitation after discharge enabled her to walk with a cane or a caster walker.

## Discussion

Incidence and clinical challenge

The reported incidence of BCIS varies in each report, but it is relatively uncommon. In addition, its mortality rate is high, and Grade 3 BCIS has been reported to have mortality rates as high as 88% [4,5]. Moreover, previous reports showed a persistently high mortality rate, even with ECMO support. In this case, the patient survived without neurological impairment, likely owing to rapid VA-ECMO initiation within 30 minutes of CPR onset, resulting in a short low-flow duration. This supports previous findings that rapid ECPR deployment is crucial for survival and neurologic preservation in perioperative cardiac arrest [6].

Pathophysiological considerations

Although the pathophysiology of BCIS is not fully elucidated, it may be caused by pulmonary microembolization, complement activation, and the release of histamine. All can lead to increased pulmonary vascular resistance (PVR) [4]. Previous studies have suggested that bone cement may induce transient right ventricular dysfunction via pulmonary vasoconstriction and ventilation-perfusion mismatch [7]. Following cementation, decreases in MAP, cardiac index, and stroke volume index, with concurrent increases in PVR, have been observed. In the present case, TEE demonstrated acute right ventricular dilatation without thromboembolic obstruction, while CT imaging revealed bone cement extending beyond the medullary cavity. This finding implies that cement extravasation into venous channels may have led to intravascular microembolization, provoking abrupt increases in PVR and right ventricular afterload. The resultant surge in pulmonary arterial pressure, compounded by pre-existing AS and PH, likely precipitated acute right ventricular failure and subsequent cardiac arrest.

Management implications

Evidence is insufficient to conclude that cement alone is the direct cause of BCIS [8]. Other factors, such as the patient's underlying conditions, surgical techniques, and intraoperative management, might be involved. Preoperative identification of high-risk patients: those with ASA-PS III-IV, chronic obstructive pulmonary disease (COPD), and diuretic or warfarin use, is critical [4]. For such patients, supplementary intraoperative preventive strategies, including medullary lavage, staged cementing techniques, and-in highly selected cases - consideration of inferior vena cava filter placement, may be considered to reduce the risk of BCIS. Importantly, meticulous intraoperative monitoring and readiness for ECPR are essential. In our case, prior interdisciplinary planning between the anesthesiology and cardiology teams enabled immediate ECMO initiation and successful resuscitation (Table 2) [2,9-15]. This experience underscores the value of preparedness, rapid decision-making, and team coordination in managing catastrophic BCIS.

**Table 2:** Summary of previous reports about BCIS in the orthopedic surgery Source: [2,9-15] * estimated by the authors based on the description in each report. BCIS, Bone cement implantation syndrome; VA-ECMO, Venoarterial extracorporeal membrane oxygenation; ROSC, Return of spontaneous circulation; CPR, Cardiopulmonary resuscitation

First Author	Year	Surgical Procedure	Outcome	Advanced Treatment	BCIS Grade	Neurological Prognosis
Govil et al. [9]	2009	2 Hip Arthroplasties, 1 Shoulder Arthroplasty, 1 Knee Arthroplasty	1 death, 3 recovered	Vasopressors, ventilation	Fatal cases: Grade 3*; Recovered cases: Grade 1, 2, 3*	Recovered cases: (1) ICU to the ward, (2) uneventful, (3) confused for 6 hours
							Mudgalkar and Ramesh [2]	2011	Cemented Hemiarthroplasty	Death	Standard CPR	Grade 3	---
							Razuin et al. [10]	2013	Total Hip Replacement	Death	Standard CPR	Grade 3^*^	---
							Izumi et al. [11]	2019	Cemented Femoral Head Hemiarthroplasty	ROSC in the operating room	Standard CPR	Grade 3^*^	With no apparent neurologic sequelae
Dradjat et al. [12]	2021	Hemiarthroplasty	ROSC in the operating room	Standard CPR	Grade 3	Without any central nervous effects to the regular ward
Zhou et al. [13]	2021	Total Hip Replacement	Recovered	Adrenaline, ventilation	Grade 2^*^	No major complications
Kumbasar and Bonde [14]	2021	Cemented Spinal Surgery	Death	VA-ECMO	Grade 3^*^	---
Valiton et al. [15]	2022	Cemented Hemiarthroplasty	Death	VA-ECMO	Grade 3	---
Kume et al. (present study)	2025	Total Hip Arthroplasty	Survive	VA-ECMO	Grade 3	Favorable

## Conclusions

This case illustrates the successful resuscitation of Grade 3 BCIS, complicated by cardiac arrest, in a patient with severe AS and moderate PH. Rapid recognition, multidisciplinary coordination, and prompt initiation of ECPR led to survival with full neurological recovery. Clinicians should maintain a high index of suspicion for BCIS and ensure perioperative readiness for extracorporeal support in high-risk orthopedic surgeries.
